# Implications of combined *NOD2* and other gene mutations in autoinflammatory diseases

**DOI:** 10.3389/fimmu.2023.1265404

**Published:** 2023-10-19

**Authors:** Hafsa Nomani, Zuoming Deng, Brianne Navetta-Modrov, Jie Yang, Mark Yun, Olga Aroniadis, Peter Gorevic, Ivona Aksentijevich, Qingping Yao

**Affiliations:** ^1^Division of Rheumatology, Allergy and Immunology, Stony Brook University Renaissance School of Medicine, Stony Brook, NY, United States; ^2^Biodata Mining and Discovery Section, National Institute of Arthritis and Musculoskeletal and Skin Diseases, Bethesda, MD, United States; ^3^Department of Family, Population and Preventive Medicine, Stony Brook University Renaissance School of Medicine, Stony Brook, NY, United States; ^4^Division of Gastroenterology and Hepatology, Stony Brook University Renaissance School of Medicine, Stony Brook, NY, United States; ^5^Inflammatory Disease Section, National Human Genome Research Institute, National Institutes of Health, Bethesda, MD, United States

**Keywords:** Nod2, NLRP3, NLRP12, autoinflammatory, familial Mediterranean fever, digenic, Yao syndrome

## Abstract

NOD-like receptors (NLRs) are intracellular sensors associated with systemic autoinflammatory diseases (SAIDs). We investigated the largest monocentric cohort of patients with adult-onset SAIDs for coinheritance of low frequency and rare mutations in *NOD2* and other autoinflammatory genes. Sixty-three patients underwent molecular testing for SAID gene panels after extensive clinical workups. Whole exome sequencing data from the large Atherosclerosis Risk in Communities (ARIC) study of individuals of European-American ancestry were used as control. Of 63 patients, 44 (69.8%) were found to carry combined gene variants in *NOD2* and another gene (Group 1), and 19 (30.2%) were carriers only for *NOD2* variants (Group 2). The genetic variant combinations in SAID patients were digenic in 66% (*NOD2/MEFV*, *NOD2/NLRP12, NOD2/NLRP3*, and *NOD2/TNFRSF1A)* and oligogenic in 34% of cases. These variant combinations were either absent or significantly less frequent in the control population. By phenotype-genotype correlation, approximately 40% of patients met diagnostic criteria for a specific SAID, and 60% had mixed diagnoses. There were no statistically significant differences in clinical manifestations between the two patient groups except for chest pain. Due to overlapping phenotypes and mixed genotypes, we have suggested a new term, “Mixed NLR-associated Autoinflammatory Disease “, to describe this disease scenario. Gene variant combinations are significant in patients with SAIDs primarily presenting with mixed clinical phenotypes. Our data support the proposition that immunological disease expression is modified by genetic background and environmental exposure. We provide a preliminary framework in diagnosis, management, and interpretation of the clinical scenario.

## Introduction

Systemic autoinflammatory diseases (SAIDs) are characterized by abnormal innate immune responses. Nucleotide-binding oligomerization domain (NOD)-like receptors (NLRs) are intracellular sensors that modulate innate immunity, and include NOD2, Pyrin, Cryopyrin, and NLRP12, NLRP1 and NLRC4 among others (1). Germline and somatic variants of NLRs are linked to polygenic and monogenic diseases, such as NOD2-associated diseases (2), and periodic fever syndromes (3). Blau syndrome or early-onset sarcoidosis (at age 4 and younger) is an autosomal dominant granulomatous disease and is caused by NOD2 mutations of high penetrance (4, 5). Another NOD2-associated disease is Yao syndrome (YAOS, OMIM #617321), formerly designated NOD2-associated autoinflammatory disease. This disease is characterized by recurrent episodes of fever, dermatitis, arthralgias, distal leg swelling, gastrointestinal complaints, sicca-like symptoms, and eyelid swelling. The specific NOD2 mutations increase susceptibility to inflammation and serve as diagnostic markers for the disease (6–8). Classical hereditary periodic fever syndromes encompass a recessively inherited Familial Mediterranean Fever (FMF; OMIM 249100), a dominantly inherited Cryopyrin-associated Periodic Syndromes (CAPS), and Tumor Necrosis Factor Receptor-associated Periodic Syndrome (TRAPS; OMIM 142680). These diseases are linked to novel and rare pathogenic missense variants that yield mutated proteins with a gain of function in various inflammatory pathways. Depending on the mutation’s impact on protein function, patients present with a spectrum of disease severity and manifestations. Patients with CAPS typically have cold-induced conjunctivitis, urticaria and arthralgia, known as Familial Cold Autoinflammatory Syndrome type 1 (FCAS1, OMIM #120100) (9), or they can present with a severe early-onset disease caused by monoallelic high-penetrance NLRP3 mutations (NOMID; OMIM 607115). However, an intermediate disease phenotype has been associated with a low-penetrance variant, Gln705Lys (Q705K), in NLRP3 (7, 8). Similar to FCAS1 in clinical phenotype, Familial Cold Autoinflammatory Syndrome type 2 (FCAS2, OMIM #611762), also called NLRP12-AID, is associated with heterozygous loss-of-function mutations in NLRP12; nearly half of patients reported to date harbor the low -penetrance NLRP12 variant, Phe402Leu (F402L) (10). TRAPS is an autosomal dominant disease characterized by recurrent fever, centrifugal rash, migratory myalgias underlying the rash, and periorbital swelling/pain; it is caused by monoallelic missense variants in the extracellular domain of *TNFR*1. The low-penetrance variant, Arg121Gln (R121Q; aka R92Q), has been reported in patients with a milder non-specific inflammatory disease (11, 12). Although these SAIDs share overlapping clinical phenotypes, they are genetically distinct and follow classical recessive or dominant mode of inheritance (13, 14).

Molecular technologies in genomic medicine, especially next-generation sequencing, are increasingly being used clinically to identify related genetic markers for an accurate diagnosis of SAIDs and other immunological diseases (15). Digenic or oligogenic inheritance of low-frequency and low-penetrance gene variants has been reported in individual patients, leading to challenges for clear interpretation of their clinical significance. We and others have previously reported cases and case series of gene variant combinations in SAID patients (14, 16). Herein, we provide detailed clinical and genetic information for the largest single-site cohort of adult patients who carry two or more variant combinations of *NOD2* and other SAID-linked genes. In conjunction with the literature, we provide the most up-to-date information on these SAIDs and genetics, and our experience in diagnosis and management.

## Materials and methods

Electronic medical records of a cohort of patients with SAIDs were retrospectively reviewed. These patients presented with a constellation of recurrent fever, rash, arthralgia, abdominal pain/diarrhea and/or chest pain among others. Patients were referred and managed by subspecialists in the Center of Autoinflammatory Disease at Stony Brook University Hospital between 2016 and April 2023. These patients were encountered after multidisciplinary care and had undergone frequently repetitive diagnostic testing. Systemic autoimmune diseases such as classic connective tissue diseases and vasculitis were ruled out; in addition, most had complete evaluations by gastroenterologists, with negative evaluations for inflammatory bowel disease (IBD). Magnetic resonance imaging of the head, echocardiography, and computerized tomography of the chest, abdomen, and pelvis were conducted if indicated. Malignant diseases were excluded. Due to unclear diagnoses and the presence of autoinflammatory clinical features, all patients underwent molecular testing including a 6-gene panel, i.e., *MEFV, TNFRSF1A, NLRP3, MVK, NLRP12*, and *NOD2* (DDC, Middlefield, Ohio, USA). A total of 44 patients were identified in our entire cohort of patients with SAIDs to carry both NOD2 and other SAID-associated gene variants (*NOD2*+other gene variants, Group 1). An individual SAID was diagnosed based on characteristic phenotype and specific genotype, as well as the classification criteria for periodic fever syndromes (17). In order to examine potential differences between patients with *NOD2* ± other gene variants, we selected typical cases of YAOS with *NOD2* variants alone (Group 2) for phenotypic comparison between the two groups. YAOS was diagnosed according to our published criteria, i.e., characteristic phenotype and specific *NOD2* variants with the exclusion of relevant diseases, such as early onset sarcoidosis or Blau syndrome (BS) and IBD (7, 18, 19). YAOS-associated *NOD2* variants are often compound heterozygous for IVS8 + 158(rs5743289, Minor Allele Frequency, MAF=0.10 in gnomAD) and another one or more *NOD2* variants, such as Arg702Trp (R702W/SNP8; rs2066804; MAF=0.025),1007fs (SNP13; rs2066847; MAF=0.015), Val955Ile (V955I; rs5743291; MAF=0.06) or rare *NOD2* variants (20). A single heterozygous *NOD2* variant, such as IVS8 + 158, V955I or rare variants are also seen.

To estimate the distribution and frequency of the combined *NOD2*+ other variant alleles identified in Group 1 patients in a control population, our collaborators at the National Institutes of Health (NIH) used the dbGaP database, the Atherosclerosis Risk in Communities (ARIC) study with dbGaP accession number phs000280.v8. p2. The ARIC study includes a cohort population and several community surveillance populations in the US. ARIC initiated community-based surveillance in 1987 for myocardial infarction and coronary heart disease incidence and mortality and created a prospective cohort of 15,792 Black and White adults ages 45 to 64 years (21). There were 2,952 unrelated individuals selected based on European-American ancestry and the availability of high-quality whole exome sequencing (WES) data. Combined gene alleles identified in the SAID patients were analyzed in the ARIC control population (Group 3). The study was approved by the Stony Brook University Institutional Research Board.

### Statistical analysis

Two-sample t-test test was used to compare continuous variables such as age between two patient groups. The Chi-square test with exact p value from Monte Carlo simulation was used to compare categorical variables such as sex. In addition, mean+/-SD were reported for continuous variables; column percentages were reported for categorical variables. Fisher’s exact test was used to compare prevalence of different genotypes between Group 1 patients and the control population, Group 3. The significance level is set at p<0.05 and all analysis was performed using SAS 9.4 (SAS Institute Inc., Cary, NC).

## Results

### The demographic, clinical, and laboratory data of patients in both groups

A total of 63 adult patients with SAIDs were included in this study, among whom 44 (69.8%) carried combined variants in two or more genes, and there were 19 (30.2%) YAOS patients with characteristic clinical features and the specific NOD2 variants. All 44 patients in Group 1 were Caucasian and 93% were female; mean age was 44 ± 13 years, and disease duration 15 ± 13 years at the time of diagnosis. The latter underscores a prolonged diagnostic delay due to lack of recognition. The demographic, clinical, and laboratory data of patients in both groups are listed (**Table 1**), and these parameters were compared between the two groups. There were no statistically significant differences between the two groups except for a higher rate of chest pain in Group 2. All patients presented with a constellation of inflammatory symptoms, including recurrent fever, rash, arthralgias/distal leg swelling, gastrointestinal complaints, sicca-like symptoms, and eyelid swelling. Other notable symptoms were myalgias, oral ulcers, chest pain/pleuritis/pericarditis, asthma, and hearing loss. Most patients (80%) reported no family history of periodic fever syndromes.

**Table 1:** Patients with *NOD2*/other gene variants vs. *NOD2* variants only.

Variable	Level	Total (N=63)	Group 1 (N=44, 66%)	Group 2** (N=19, 34%)	P-value*
Age at diagnosis (year)		43 ± 13	44 ± 13	41 ± 14	0.5057
Disease duration at diagnosis (year)		13 ± 12	15 ± 13	9 ± 10	0.0682
Sex	Female	59 (94%)	41 (93%)	18 (95%)	1.0000
Race	Caucasian	63 (100%)	44 (100%)	19 (100%)	.
Fatigue	Yes	60 (95%)	42 (95%)	18 (95%)	1.0000
Night sweats	Yes	13 (21%)	11 (25%)	2 (11%)	0.3072
Headaches	Yes	31 (49%)	23 (52%)	8 (42%)	0.4588
Fever	Yes	42 (67%)	29 (66%)	13 (68%)	0.8461
Skin rash	Yes	58 (92%)	41 (93%)	17 (89%)	1.0000
Arthralgia	Yes	50 (79%)	33 (75%)	17 (89%)	0.3074
Lower extremity swelling	Yes	36 (57%)	22 (50%)	14 (74%)	0.0813
Myalgia	Yes	27 (43%)	19 (43%)	8 (42%)	0.9368
Oral ulcer	Yes	25 (40%)	18 (41%)	7 (37%)	0.7620
Gastrointestinal symptoms	Yes	54 (86%)	37 (84%)	17 (89%)	0.7056
Pain	Yes	47 (75%)	31 (70%)	16 (84%)	0.3537
Diarrhea	Yes	43 (68%)	30 (68%)	13 (68%)	0.9851
Dry eyes and mouth	Yes	42 (67%)	30 (68%)	12 (63%)	0.6979
Eyelid swelling	Yes	29 (46%)	20 (45%)	9 (47%)	0.8888
Hearing loss/decrease	Yes	15 (24%)	10 (23%)	5 (26%)	0.7589
Chest pain	Yes	18 (29%)	9 (20%)	9 (47%)	0.0300
Pleuritis	Yes	2 (3%)	1 (2%)	1 (5%)	1.0000
Pericarditis	Yes	7 (11%)	3 (7%)	4 (21%)	0.1911
Asthma	Yes	17 (27%)	12 (27%)	5 (26%)	0.9374
Proteinuria/hematuria	Yes	3 (5%)	3 (7%)	0 (0%)	0.5523
Raised ESR/CRP/ferritin	Yes	21 (33%)	12 (27%)	9 (47%)	0.1204

* For continuous variables, p-values were based on t-tests; for categorical variables, p-values were based on Monte Carlo simulation from a Chi-squared test.

mean+/-SD were reported for continuous variables; column percentages were reported for categorical variables.

** Patients with NOD2 IVS8 + 158/R702W (6), NOD2 IVS8 + 158/1007fs (5), NOD2 IVS8 + 158 (5), NOD2 IVS8 + 158/Q908R (1), NOD2 IVS8 + 158/N852S (1), and NOD2 Asp925Gly, all being heterozygous.

### Genotyping results of patients in both groups and control population

Of the 44 SAID patients in Group 1, all patients carried *NOD2* monoallelic or biallelic variants, as well as other gene variants. Most patients carried digenic variants, while oligogenic variants were found in a minority of patients. Among the digenic variants were *NOD2/MEFV, NOD2/NLRP12, NOD2/NLRP3*, and *NOD2/TNFRSF1A* in descending order of frequency. The oligogenic variants were *NOD2/NLRP3/NLRP12, NOD2/MEFV/NLRP12, NOD2/MEFV/TNFRSF1A, NOD2/NLRP12/TNFRSF1A*, and *NOD2/MEFV/NLRP3* (**Table 2**). Most gene combinations were composed of low frequency/penetrance and rare variants, with the digenic variants in *NOD2* and *MEFV* being the most common. To compare the distribution and frequency of these combined SAID-associated genetic variants in patients (Group 1) with those in the control population (Group 3), we used and analyzed the WES data of 2,952 subjects with European-American ancestry (**Table 2**). The combined gene variants identified in patients (Group 1) were either absent (23/44) or significantly lower in the ARIC control population. All 19 patients in Group 2 carried *NOD2* variants, compound heterozygote mostly.

**Table 2:** Summary of genotyping results of patients in group 1 and ARIC control *.

Genotypes	Diagnosis	Group 1: NOD2 and other gene variants (N=44), n (%)	Group 3(Control): dbGap (ARIC) (N=2, 952), n (%)	P-value**
NOD2 and MEFV				
NOD2 IVS8 + 158 + MEFV E148Q	YAOS, FMF	2 (4.55)	7 (0.23)	0.0071
NOD2 IVS8 + 158/1007fs + MEFV I591M	YAOS	1 (2.27)	0(0)	0.0147
NOD2 A725G + MEFV E148Q	FMF	1 (2.27)	0 (0)	0.0147
NOD2 D154N + MEFV K695R	YAOS	1 (2.27)	0 (0)	0.0147
NOD2 IVS8 + 158 + MEFV E148Q, P369S, R408Q	FMF	1 (2.27)	1 (0.03)	0.0292
NOD2 IVS8 + 158/1007fs + MEFV M694V	Mixed	1 (2.27)	0 (0)	0.0147
NOD2 IVS8 + 158/R791Q + MEFV E148Q	YAOS	1 (2.27)	7 (0.23)	0.0071
NOD2 IVS8 + 158/R702W/V955I + MEFV R501H	YAOS	1 (2.27)	0 (0)	0.0147
NOD2 G908R/V955I + MEFV A744S	YAOS	1 (2.27)	0 (0)	0.0147
NOD2 1007fs+MEFV P369S, R408Q	Mixed	1 (2.27)	0 (0)	0.0147
NOD2 and NLRP12				
NOD2 1007fs + NLRP12 Y618X	YAOS	1 (2.27)	0 (0)	0.0147
NOD2 P668L + NLRP12 F402L	Mixed	1 (2.27)	0 (0)	0.0147
NOD2 IVS8 + 158/R702W + NLRP12 F402L, NOD2 IVS8 + 158 +NLRP12 F402L	Mixed	3 (4.55)	85 (2.88)	0.1374
NOD2 IVS8 + 158 + NLRP12 G921R	YAOS	1 (2.27)	0 (0)	0.0147
NOD2 IVS8 + 158/R702W + NLRP12 T1043I	Mixed	1 (2.27)	0 (0)	0.0147
NOD2 R87C + NLRP12 F402L	YAOS	1 (2.27)	0 (0)	0.0147
NOD2 IVS8 + 158/1007fs + NLRP12 F402L	Mixed	2 (4.55)	82 (2.78)	0.3512
NOD2 and NLRP3				
NOD2 IVS8 + 158/R702W + NLRP3 V200M	YAOS	1 (2.27)	9 (0.30)	0.1377
NOD2 IVS8 + 158 + NLRP3 Q705K	Mixed	1 (2.27)	33 (1.11)	0.397
NOD2 IVS8 + 158/R702W + NLRP3 Q705K	YAOS	1 (2.27)	51 (1.72)	0.5398
NOD2 V955I + NLRP3 V200M	Mixed	1 (2.27)	5 (0.17)	0.0850
NOD2 N289S/V955I + NLRP3 Q705K	YAOS	1 (2.27)	22 (0.75)	0.2893
NOD2 and TNFRSF1A				
NOD2 IVS8 + 158/1007fs + TNFRSF1A R92Q	Mixed	1 (2.27)	12 (0.41)	0.1753
NOD2 IVS8 + 158/R702W + TNFRSF1A R92Q	YAOS	1 (2.27)	17 (0.57)	0.2344
NOD2 S431L/V793M + TNFRF1A R92Q	Mixed	1 (2.27)	0 (0)	0.0147
Oligogenic variants				
NOD2 IVS8 + 158(homozygous)/R702W + MEFV P365S/R408Q +TNFRSF1A N145S	YAOS	1 (2.27)	0 (0)	0.0147
NOD2 V955I + MEFV K695R + NLRP3 Q705K	Mixed	1 (2.27)	0 (0)	0.0147
NOD2/NLRP3/NLRP12				
NOD2 IVS8 + 158/V955I + NLRP3 Q705K + NLRP12 G448A	Mixed	1 (2.27)	0 (0)	0.0147
NOD2 R702W/V955I + NLRP3 Q705K + NLRP12 F402L	YAOS	1 (2.27)	2 (0.06)	0.0434
NOD2 IVS8 + 158/R702W + NLRP3 V643M + NLRP12 F402L	Mixed	1 (2.27)	0 (0)	0.0147
NOD2/MEFV/NLRP12				
NOD2 IVS8 + 158(homozygous) + MEFV I591M + NLRP12 F402L	YAOS	1 (2.27)	0 (0)	0.0147
NOD2 IVS8 + 158 + MEFV R329H + NLRP12 G448A	Mixed	1 (2.27)	0 (0)	0.0147
NOD2 G908R + MEFV A744S + TNFRSF1A R92Q	Mixed	2 (4.55)	0 (0)	0.0002
NOD2V955I + MEFV K695R + TNFRSF1A R92Q	Mixed	1 (2.27)	1 (0.03)	0.0292
NOD2 IVS8 + 158 + NLRP12 F402L + TNFRSF1A R92Q	Mixed	1 (2.27)	4 (0.14)	0.0714
NOD2 IVS8 + 158/R702W + MEFV V726A + NLRP3 Q705K	Mixed	1 (2.27)	0 (0)	0.0147
NOD2 IVS8 + 158 + MEFV V726A + NLRP3 Q705K	Mixed	1 (2.27)	0 (0)	0.0147
NOD2 R791Q + MEFV I591T + NLRP3 V200M + TNFRSF1A R92Q	Mixed	1 (2.27)	0 (0)	0.0147
NOD2IVS8 + 158(homozygous)/1007fs + NLRP3 Q705K + MEFV E148Q/P365S/R408Q	Mixed	1 (2.27)	0 (0)	0.0147

*All variants are heterozygous unless indicated otherwise.

**P-values were based on two-sided Fisher’s exact test.

### Diagnostic challenge

Clinical phenotypes with features suggestive of autoinflammatory disease were found for all patients at presentation. Following detailed phenotypic evaluations and phenotype-genotype correlations, 16/44(36%) of patients in Group 1 were diagnosed as YAOS, 3/44(6.8%) as atypical FMF, and the remaining with mixed diagnoses of two or more SAIDs, such as YAOS, FMF, NLRP3-AID, NLRP12-AID, and TRAPS. Having *NOD2* as the denominator in all patients with combined variants, we asked if there were similarities between these patients with *NOD2* ± other gene variants. Our results demonstrated no statistically significant differences in demographics, clinical phenotypes and laboratory results between the two groups except for lower frequency of chest pain in Group 1 (**Table 1**), suggesting similar but mixed clinical phenotypes among patients with *NOD2* ± other gene variants. No significant internal solid organ damage or dysfunction was found for either group. However, some patients in both groups experienced frequent disease flares over a prolonged period of time, resulting in impaired ability to function physically and mentally. Nearly half of the patients in either group received IL-1 inhibitors (Canakinumab or Anakinra), many after trials of colchicine or sulfasalazine, with good response.

## Discussion

### Potential significance of combined gene variants

SAIDs are generally associated with variants in a single gene locus, though variant combinations in two or more relevant genes can be identified in individual patients as in our study. As a result, challenges arise for diagnosis and management. In the current study, genetic variants classified as variants of uncertain significance in *NOD2* and *MEFV* genes based on the Infevers database were found to be the most frequently inherited https://infevers.umai-montpellier.fr/web/index.php.

What could be the clinical significance of combined variants in individual SAID patients who present with adult-onset disease? We did not observe significant differences between the groups in clinical phenotypes, which likely extends to disease course and therapy. As seen in **Table 1**, YAOS was diagnosed more frequently among patients with combined *NOD2* and other SAID gene variants, and the majority of patents with YAOS carried compound *NOD2* variants or rare variants. This might indicate more influences of the NOD2 variants on phenotypic expression in the genetic background containing multiple SAID genes. We stratified patients in both groups into subgroups: YAOS patients with *NOD2* variants only (n=19), patients diagnosed with YAOS and *NOD2*/other gene variants (n=16), and patients with mixed or other diagnoses (n=28). Further analyses showed statistically significant differences between YAOS patients (n=35) vs patients with mixed or other diagnoses (n=28). Lower extremity swelling as a characteristic finding for YAOS was significantly higher in YAOS patients (69%) than patients with mixed or other diagnoses (43%, P value=0.0404), whereas headache was significantly higher in patients with mixed or other diagnoses (**Table 3**, Supplemental). In addition, chest pain was significantly higher in YAOS patients with NOD2 variants only than those with NOD2+other gene variants (**Table 4**, Supplemental). These data indicate that patients with *NOD2*+other gene variants may not have more severe diseases and poorer outcomes than those with variants in a single gene locus. Another possibility is that combined variants could be synergistic, antagonistic, or perhaps both, depending on the type of gene combinations.

**Table 3:** YAOS patients versus patients with mixed or other diagnoses.

Variable	Level	Total (N=63)	YAOS patients (N=35, 56%)	Patients with mixed or other diagnoses (N=28, 44%)	P-value*
Age at diagnosis (year)	35 vs 28	44 ± 21	45 ± 19	43 ± 27	0.9283
Disease duration at diagnosis (year)	35 vs 28	8 ± 17	6 ± 18	11 ± 17	0.2440
Sex	Female	59 (94%)	33 (94%)	26 (93%)	1.0000
	Male	4 (6%)	2 (6%)	2 (7%)	
Race	Caucasian	63 (100%)	35 (100%)	28 (100%)	.
Fatigue	Yes	60 (95%)	34 (97%)	26 (93%)	0.5785
Night sweats	Yes	13 (21%)	6 (17%)	7 (25%)	0.4438
Headaches	Yes	31 (49%)	13 (37%)	18 (64%)	0.0322
Fever	Yes	42 (67%)	22 (63%)	20 (71%)	0.4733
Skin rash	Yes	58 (92%)	31 (89%)	27 (96%)	0.3722
Arthralgia	Yes	50 (79%)	31 (89%)	19 (68%)	0.0618
Lower extremity swelling	Yes	36 (57%)	24 (69%)	12 (43%)	0.0404
Myalgia	Yes	27 (43%)	16 (46%)	11 (39%)	0.6084
Oral ulcer	Yes	25 (40%)	12 (34%)	13 (46%)	0.3276
Gastrointestinal symptoms	Yes	54 (86%)	30 (86%)	24 (86%)	1.0000
Pain	Yes	47 (75%)	28 (80%)	19 (68%)	0.2712
Diarrhea	Yes	43 (68%)	23 (66%)	20 (71%)	0.6283
Dry eyes and mouth	Yes	42 (67%)	24 (69%)	18 (64%)	0.7199
Eyelid swelling	Yes	29 (46%)	16 (46%)	13 (46%)	0.9549
Hearing loss/decrease	Yes	15 (24%)	9 (26%)	6 (21%)	0.6915
Chest pain	Yes	18 (29%)	11 (31%)	7 (25%)	0.5746
Pleuritis	Yes	2 (3%)	2 (6%)	0 (0%)	0.4952
Pericarditis	Yes	7 (11%)	5 (14%)	2 (7%)	0.4463
Asthma	Yes	17 (27%)	10 (29%)	7 (25%)	0.7510
Proteinuria/hematuria	Yes	3 (5%)	1 (3%)	2 (7%)	0.5868
Raised ESR/CRP/ferritin	Yes	21 (33%)	12 (34%)	9 (32%)	0.8577
Hypogammaglobinemia	Yes	5 (8%)	4 (11%)	1 (4%)	0.3714

*For continuous variables, p-values were based on wilcoxon’s rank sum test; for categorical variables, p-values were based on Monte Carlo simulation from a Chi-squared test.

median+/-IQR were reported for continuous variables; column percentages were reported for categorical variables.

**Table 4:** YAOS patients with variants of NOD2± other genes.

Variable	Level	Total (N=35)	NOD2 gene only (N=19, 54%)	NOD2+other genes (N=16, 46%)	P-value*
Age at diagnosis (year)	19 vs 16	45 ± 19	44 ± 23	46 ± 18	0.4657
Disease duration at diagnosis (year)	19 vs 16	6 ± 18	5 ± 10	14 ± 18	0.3188
Sex	Female	33 (94%)	18 (95%)	15 (94%)	1.0000
	Male	2 (6%)	1 (5%)	1 (6%)	
Race	Caucasian	35 (100%)	19 (100%)	16 (100%)	.
Fatigue	Yes	34 (97%)	18 (95%)	16 (100%)	1.0000
Night sweats	Yes	6 (17%)	2 (11%)	4 (25%)	0.3815
Headaches	Yes	13 (37%)	8 (42%)	5 (31%)	0.5079
Fever	Yes	22 (63%)	13 (68%)	9 (56%)	0.4579
Skin rash	Yes	31 (89%)	17 (89%)	14 (88%)	1.0000
Arthralgia	Yes	31 (89%)	17 (89%)	14 (88%)	1.0000
Lower extremity swelling	Yes	24 (69%)	14 (74%)	10 (63%)	0.4777
Myalgia	Yes	16 (46%)	8 (42%)	8 (50%)	0.6405
Oral ulcer	Yes	12 (34%)	7 (37%)	5 (31%)	0.7284
Gastrointestinal symptoms	Yes	30 (86%)	17 (89%)	13 (81%)	0.6430
Pain	Yes	28 (80%)	16 (84%)	12 (75%)	0.6819
Diarrhea	Yes	23 (66%)	13 (68%)	10 (63%)	0.7131
Dry eyes and mouth	Yes	24 (69%)	12 (63%)	12 (75%)	0.4983
Eyelid swelling	Yes	16 (46%)	9 (47%)	7 (44%)	0.8305
Hearing loss/decrease	Yes	9 (26%)	5 (26%)	4 (25%)	1.0000
Chest pain	Yes	11 (31%)	9 (47%)	2 (13%)	0.0373
Pleuritis	Yes	2 (6%)	1 (5%)	1 (6%)	1.0000
Pericarditis	Yes	5 (14%)	4 (21%)	1 (6%)	0.3548
Asthma	Yes	10 (29%)	5 (26%)	5 (31%)	0.7475
Proteinuria/hematuria	Yes	1 (3%)	0 (0%)	1 (6%)	0.4622
Raised ESR/CRP/ferritin	Yes	12 (34%)	9 (47%)	3 (19%)	0.1562
Hypogammaglobinemia	Yes	4 (11%)	2 (11%)	2 (13%)	1.0000

*For continuous variables, p-values were based on wilcoxon’s rank sum test; for categorical variables, p-values were based on Monte Carlo simulation from a Chi-squared test.

median+/-IQR were reported for continuous variables; column percentages were reported for categorical variables.

### Molecular pathways underlying combined gene expression

**Figure 1:**
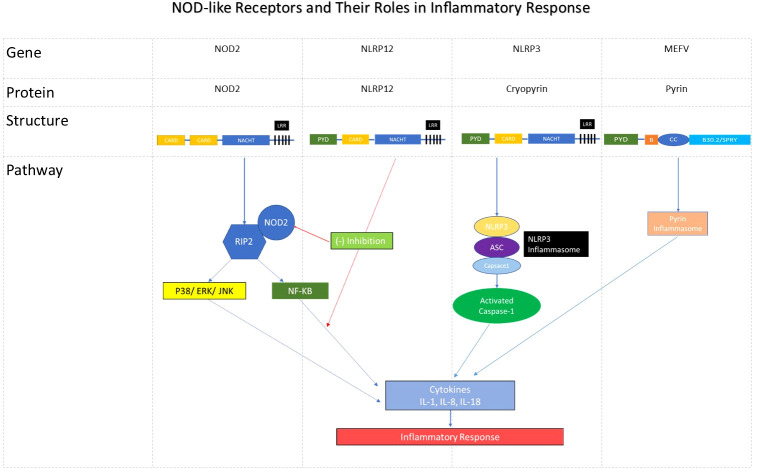
NOD-like receptors and their roles in inflammatory response. The diagram shows the names of respective NLR gene/encoded protein and its structure. These proteins give rise to inflammatory response via NOD2/RIPK2/NF-kB and other pathways involving the NLRP3- and Pyrin inflammasomes. NLRP12 can inhibit RIPK2/NOD2 complex and NF-kB. Note: CARD, Caspase-recruiting domain; LRR, Leucine rich repeat; RIPK2, Receptor interacting protein kinase; P38, 3 Mitogen activated protein kinases (MAPKs); ERK, extracellular signal-regulated kinase (ERK); JNK, c-JUN N terminal kinase (JNK); ASC, Apoptosis-associated speck-like protein containing a CARD.

To understand the role of these intracellular sensors in autoinflammatory diseases, individual genes(*NOD2, NLRP3, NLRP12*, and *MEFV*) and their downstream pathways are schematically depicted in **Figure 1** based on literature review (1, 2). NLR interactions are complex, and NOD2 may function in concert with other immune sensors to regulate immune and inflammatory responses in different tissues. NOD2 shares a similar molecular structure with NLRP3 and NLRP12, and has an important role with regard to innate immune responses in gut. NOD2 recognizes a bacterial wall component, Muramyl Dipeptide (MDP), and functions in defense against microbial infection, in the regulation of the inflammatory process, and apoptosis (2). *NOD2* mutations are linked to Crohn’s disease (40% patients), Blau syndrome, and YAOS. NOD2 signaling pathway involves receptor interacting protein kinase 2 (RIP2) that can be activated dependently on or independently of NOD2 (22). There is a cross-talk between NOD2 and Toll-like receptors (TLRs). NOD2 activation causes interferon regulatory factor 4(IRF4) expression, which in turn binds to tumor necrosis factor receptor associated factor 6(TRAF6) and RIP2, leading to NF-kB activation (23, 24). NOD2 mutations have been recently shown to cause loss of NOD2 cross-regulatory function involving IRF4 (25). In murine models, NOD2, together with NLRP3, caspase-1, Apoptosis-associated Speck-like protein containing a Caspase activation and recruitment domain (ASC), and RIP2, is required for MDP-induced IL-1 release (26). The NLRP3 inflammasome mediates intestinal inflammation in NOD2-deficient mice (27). NLRP3 also interacts with IRF4 (28).The function of NLRP12 is yet unclear in humans. In mice, NLRP12 has been shown to play a role in the proteasomal degradation of NOD2 and to promote bacterial tolerance and colonization by enteropathogens. NLRP12 suppresses MDP-induced NF-κB activation by targeting the NOD2/RIP2 complex. MDP tolerance is lost in murine monocytes deficient for NLRP12 (29, 30). There may be indirect interactions between NLRP12 and IRF4 (31). MEFV gene is an immediate early gene for interferon-gamma (32). These data suggest cooperative and interrelated roles of some NLRs in disease, and that IRF4 could be a key transcription factor for the orchestration of NLR functions in immune cells. Further study of the role of IRF4 within the context of NLR interactions in autoinflammatory disease is needed. In addition, we previously reported a combination of NOD2 and UBA1 mutations in a patient with an autoinflammatory disease and VEXAS syndrome, noting that both genes are involved in, or regulated by, the ubiquitin pathway (33). Taken together, our study has extended the understanding of the enormous complexity of the genetic influences that underlie autoinflammatory diseases whose clinical characteristics are often superimposable. In other words, similar clinical phenotypes in SAIDs may be caused in different genetic background, suggesting the biological complexity behind the disease phenotypes. Based on our study results and the literature data, it would be reasonable to perform higher-order molecular testing, as first-level testing may not suffice to reveal the underlying genetic mechanisms.

### Implication of low frequency and low penetrance variants in SAIDs

The majority of patients with combined gene variants carried both low-frequency/low- penetrance and rare variants. These variants are *MEFV* Glu148Gln (E148Q; rs3743930; MAF=0.07 in gnomAD, as high as 0.30 in Asian populations), *NLRP3* Gln705Lys (Q705K; rs35829419; MAF=0.038), *NLRP12* Phe402Leu (F402L; rs199985574; MAF=0.05), and *TNFRSF1A* Arg121Gln (R92Q; rs4149584; MAF=0.012), and selected *NOD2* variants. These previously reported risk alleles have been associated with susceptibility to SAIDs or they play a role in modifying disease expression (10, 12, 30, 34–37). Notably, these low-frequency variants were found to be concurrent with rare ones in most patients. Furthermore, the distribution and frequency of these variant combinations in patients were either absent or significantly lower in the control population. These data suggest that these variant combinations are clinically significant. Genetic variants contributing to disease lie on a spectrum from rare alleles with large effect sizes to more common alleles with small effect sizes. There is a “gray zone” between these two extremes, as in this study, which is poorly defined with regard to terminology, classification and clinical reportability (38). These autoinflammatory diseases with low penetrance variants have been recently classified as the new category of “Genetically Transitional Disease” (GTD). GTD straddles the old distinction between monogenic and polygenic, where a large-effect mutation is necessary, but not sufficient, to cause disease (39). GTD emphasizes the key role of genetic background in modifying both penetrance and expressivity of a mutation or variant. This concept may also be important for the genetic counseling of these patients, supplementing the traditional interpretation of monogenic autosomal dominant or recessive diseases.

Genome-Wide Association Studies (GWAS) have revealed thousands of genetic variants associated with hundreds of human diseases. However, those that reach genome-wide statistical significance explain only a small fraction of heritability. Common variants may explain more than 50% heritability of many common diseases, including Crohn’s disease, type I diabetes, and multiple sclerosis (40). Overall, the composite effect of variants of small effect may have equal or greater impact than a rare pathogenic mutation with a large effect for more common human phenotypes (41). This concept and theory may in part help explain the implication of our findings, i. e., the coexistence of low penetrance variants in individual patients. Our data also suggest that a combination of genetic defects in different genes, which converge to a common pathogenic pathway, may have a synergistic effect and predispose individuals to SAIDs. For example, CAPS, NLRP12-AID and TRAPS have been classified as IL-1 mediated autoinflammatory diseases based on the patients’ response to IL-1 inhibitors (42, 43). The variant combinations in our study also support the conventional wisdom that genetic background influences both penetrance and phenotypic expressivity of gene mutations (39). Genetic background refers to all other related genes that may interact with the gene of interest to potentially influence specific phenotype in concert with environment (44). Our data may also serve as an example to understand the interactions between genes of interest, genetic background, and environmental or other factors in genetic diseases. Typical monogenic diseases are autosomal dominant disease (Huntington’s disease, HD) and recessive disease (Cystic fibrosis, CF). In these Mendelian diseases, there are slight but not predominant differences in female: male ratio, 54.5%/45.5% for HD (45), and 47.1%/52.9% for CF (46). One explanation is that primary mutations are highly penetrant and major players in these diseases. The candidate gene interactions with genetic background contribute to disease. For example, primary CFTR gene interacts with its modifier gene in the background contribute to CF (47). Unlike monogenic diseases, diseases like YAOS are mostly associated with reduced penetrance variants and are considered as GTD. Based on the GTD model of necessity and insufficiency, the candidate gene, for example, NOD2, genetic background containing certain SAID genes, and other factors such as sex hormones could play more important roles in the disease. Hormonal imbalance is known to cause inflammation or immune response (48), whereas androgen acts via its receptor on macrophages to suppress inflammation or immune response (24). Consequently, this could skew towards female predominance in the disease. Concretely, *NOD2* variants are the denominator in all combinations of variants in our study; if *NOD2* is considered as the candidate gene, other gene variants such as *MEFV*, *NLRP3, NLRP12* and*TNFRSF1A* may be entertained as modifying alleles within genetic background. As noted in **Table 3**, among the overwhelming majority of 44 patients with combined variants, each carried a different combination of a limited number of the SAID genes to constitute a different but related genetic background, as analogous to the ten Arabic numerals from 0 to 9 for writing numbers or codes. Although most *NOD2* variants are low-penetrant, their effect could be upwardly influenced by additional germline genetic alleles (39). As most of these gene variants are gain-of-function, they might have been under a positive evolutionary selection as they could provide better immune responses against various pathogens (49).Future genome-based studies in large cohorts of patients may help identify more SAIDs-associated modifying alleles. Acquired somatic mutations in the same genes may further contribute to disease expressivity in adult-onset autoinflammatory and autoimmune diseases.

### Digenic/oligogenic disease: conceptual utility in SAIDs

The term “Digenic disease” refers to combinations of variants in two genes (50), and encompasses disorders in which both genes are required for expression or situations where a modifier gene significantly influences phenotype. The term “Oligogenic disease” refers to variant combinations in multiple genes. Based on these concepts, variant combinations in our study may be classified as “Digenic” in the majority of cases and “Oligogenic” in the minority. These variant combinations in our patients may be significant for the following reason. FMF is generally classified as a monogenic recessive disease, caused by biallelic missense gain-of-function mutations in *MEFV*. However, approximately 25% of FMF patients only carry monoallelic *MEFV* mutations (51). In fact, several studies have shown the monoallelic association with FMF (52–55), suggesting it is a dominant disease in some cases, where GTD model may apply. In our study, most patients with *NOD2/MEFV* variants were heterozygous for *MEFV* E148Q. Several studies of its pathogenic role in classic FMF were conducted with mixed results (35), but most literature data have favored its contributory role in autoinflammatory phenotypes that may or may not be classified as typical FMF as is the case with two recent independent Turkish studies (36, 56). An Israeli study showed that *MEFV* E148Q is likely a contributory genetic factor when coinherited with M694V (35). Based on our current study, we would assume that patients with carriage of both *NOD2* and heterozygous *MEFV* mutations could mimic biallelic compound heterozygotes of *MEFV* mutations leading to autoinflammatory diseases with mixed phenotypic expressivity. This could explain a proportion of FMF patients with monoallelic *MEFV* mutations.

In our study, approximately 20% of patients reported a family history of similar symptoms. A co-segregation analysis of three families in our cohort found that symptomatic relatives shared digenic or oligogenic variants with the probands. Functional studies of some individual low penetrance variants involved in our study were previously conducted by others with ambigous results, specifically in regard to the *MEFV* E148Q variant. Cells expressing *NLRP3* Q705K have mildly increased caspase 1 activity and cleavage, and such patients responded to an IL-1 inhibitor therapy (34). In another study, human monocytic cell lines transduced with Q705K produced significantly higher level of IL-1β and IL-18 than wild type, indicating a gain-of-function (57). In addition, we previously demonstrated abnormal NOD2 expression, NOD2 pathway activation, and a cytokine profile in patients harboring *NOD2* variants, IVS8 + 158 and R702W (58).

### Mixed NLR-associated autoinflammatory disease

Genotype-phenotype correlation may be readily apparent in patients with monoallelic or biallelic variants in a single gene, but can be challenging concerning combined variants from different genes. Additionally, *NOD2* was the denominator in all the combined variants, and our study indicates that these NOD2 variants together with other relevant SAID-associated gene variants contribute to disease pathogenesis. We, therefore, have suggested a new term at the American College of Rheumatology Annual Meeting in 2022, mixed NLR-associated Autoinflammatory Disease (NLR-AID) to denote NLR involvement in the mixed diagnosis, which could be assigned an ICD10 code in the future for insurance billing and research purposes. Biologic therapy with IL-1 inhibitors are generally effective for mixed NLR-AID, YAOS, as well as hereditary periodic fever syndromes (59, 60). One would ask whether mixed NLR-AID could represent an independent entity that might be caused by a hidden pathogenic mutation. To clarify about it, whole exome or genome sequencing would be needed.

In conclusion, this is the largest single site-cohort of autoinflammatory disease patients with *NOD2*+ other gene variants. Most patients underwent a diagnostic odyssey during a prolonged “mysterious” illness. Unlike many common diseases for which there are readily available guidelines or consensus, SAIDs are rare diseases, and best evidence may come from case reports and case series (61). We provide rational interpretations and our experience with regard to diagnosis, classification, and management.

### Limitations

This is a single center study with benefit of uniformity and standardization of the study population. Relative to studies of common diseases, the sample size of our current study is small because the disease entity and associated clinical scenario is rare. We hope that more studies using similar cohorts of patients with these combined gene variants should be performed to replicate our findings.

## Data availability statement

The datasets presented in this study can be found in online repositories. The names of the repository/repositories and accession number(s) can be found in the article/supplementary material.

## Ethics statement

The studies involving humans were approved by the Institutional Review Board of Stony Brook University. The studies were conducted in accordance with the local legislation and institutional requirements. The ethics committee/institutional review board waived the requirement of written informed consent for participation from the participants or the participants’ legal guardians/next of kin because of retrospective review of electronic medical records.

## Author contributions

HN: Data curation, Formal Analysis, Investigation, Resources, Writing – review and editing. ZD: Data curation, Investigation, Resources, Writing – review and editing, Methodology. BN: Investigation, Resources, Writing – review and editing. JY: Resources, Writing – review and editing, Data curation. MY: Resources, Writing – review and editing, Investigation. OA: Resources, Writing – review and editing. PG: Resources, Writing – review and editing. IA: Writing – review and editing, Data curation, Formal Analysis, Investigation. QY: Data curation, Formal Analysis, Investigation, Writing – review and editing, Conceptualization, Methodology, Project administration, Resources, Supervision, Validation, Writing – original draft.
